# Placental endoplasmic reticulum stress negatively regulates transcription of placental growth factor via ATF4 and ATF6β: implications for the pathophysiology of human pregnancy complications

**DOI:** 10.1002/path.4678

**Published:** 2016-01-12

**Authors:** Masahito Mizuuchi, Tereza Cindrova‐Davies, Matts Olovsson, D Stephen Charnock‐Jones, Graham J Burton, Hong Wa Yung

**Affiliations:** ^1^ Centre for Trophoblast Research, Department of Physiology, Development, and NeuroscienceUniversity of CambridgeUK; ^2^ Department of Women's and Children's HealthUppsala UniversitySweden; ^3^ Department of Obstetrics and GynaecologyUniversity of Cambridge, The Rosie HospitalCambridgeUK; ^4^ National Institute for Health ResearchCambridge Comprehensive Biomedical Research CentreCambridgeUK

**Keywords:** placental growth factor, pre‐eclampsia, endoplasmic reticulum stress, placenta, pregnancy and reproduction

## Abstract

Low maternal circulating concentrations of placental growth factor (PlGF) are one of the hallmarks of human pregnancy complications, including fetal growth restriction (FGR) and early‐onset pre‐eclampsia (PE). Currently, PlGF is used clinically with other biomarkers to screen for high‐risk cases, although the mechanisms underlying its regulation are largely unknown. Placental endoplasmic reticulum (ER) stress has recently been found to be elevated in cases of FGR, and to an even greater extent in early‐onset PE complicated with FGR. ER stress activates the unfolded protein response (UPR); attenuation of protein translation and a reduction in cell growth and proliferation play crucial roles in the pathophysiology of these complications of pregnancy. In this study, we further identified that ER stress regulates release of PlGF. We first observed that down‐regulation of PlGF protein was associated with nuclear localization of ATF4, ATF6α and ATF6β in the syncytiotrophoblast of placentae from PE patients. Transcript analysis showed a decrease of PlGF mRNA, and an increase from genes encoding those UPR transcription factors in placentae from cases of early‐onset PE, but not of late‐onset (>34 weeks) PE, compared to term controls. Further investigations indicated a strong correlation between ATF4 and PlGF mRNA levels only (r = − 0.73, p < 0.05). These results could be recapitulated in trophoblast‐like cells exposed to chemical inducers of ER stress or hypoxia–reoxygenation. The stability of PlGF transcripts was unchanged. The use of small interfering RNA specific for transcription factors in the UPR pathways revealed that ATF4 and ATF6β, but not ATF6α, modulate PlGF transcription. To conclude, ATF4 and ATF6β act synergistically in the negative regulation of PlGF mRNA expression, resulting in reduced PlGF secretion by the trophoblast in response to stress. Therefore, these results further support the targeting of placental ER stress as a potential new therapeutic intervention for these pregnancy complications. © 2015 The Authors. *Journal of Pathology* published by John Wiley & Sons Ltd on behalf of Pathological Society of Great Britain and Ireland.

## Introduction

Pre‐eclampsia and fetal growth restriction (FGR) are two major causes of neonatal mortality and morbidity 1. Many potential causes have been proposed, although the pathological mechanisms underlying these disorders are still not fully understood. Hence, effective treatments remain elusive 2, 3. Pre‐eclampsia is a multi‐system, heterogeneous syndrome and increasingly two forms are recognized, early‐ and late‐onset 4, 5. Roberts and Redman stated in 1993 that PE 'starts with the placenta and ends with the endothelium' 6. This hypothesis has been developed further 7, but remains the mainstay of current understanding of the pathophysiology. The early‐onset form is commonly associated with growth restriction and severe placental pathology. A failure in spiral artery remodelling is thought to lead to poor uteroplacental perfusion and intermittent placental hypoxia. Consequently, the placenta suffers oxidative stress, causing the release of factors that have systemic actions on various maternal organs. By contrast, spiral arterial and placental pathology are minimal in late‐onset pre‐eclampsia. It is thought that in these cases the mother carries a genetic predisposition to cardiovascular disease that renders her endothelium hypersensitive to factors released from a relatively normal placenta. Since maternal endothelial cell activation appears to be the common end‐point that unifies various features of the syndrome, attention has focused on an imbalance between angiogenic and anti‐angiogenic factors in the maternal circulation as one of the underlying mechanisms 2, 8.

The pro‐angiogenic factor, placental growth factor (PlGF), is a homodimeric glycoprotein with significant homology to vascular endothelial growth factor A (VEGF‐A), which was originally identified in the human placenta 9. It is an important regulator of angiogenesis and vasculogenesis 10 and four variants, −1, −2, −3 and −4, have been identified so far. All are primarily expressed in the placenta, predominately localized to the syncytiotrophoblast and endothelial cells 11, 12, 13, 14, 15. PlGF‐1 and PlGF‐3 are secreted forms, whereas PlGF‐2 and PlGF‐4 are membrane‐bound because of their heparin‐binding domains. PlGF binds to VEGF receptor 1 (VEGFR1), also known as Flt‐1, thereby initiating kinase mediated signalling 16.

In cases of FGR and pre‐eclampsia, maternal circulating PlGF concentrations are reduced 17, 18, particularly in early‐onset pre‐eclampsia. Levels of other factors in the angiogenic balance are also altered. Anti‐angiogenic factors, including the soluble Fms‐like tyrosine kinase‐1 receptor (sFlt‐1) that binds VEGF‐A and soluble endoglin (sEng), are increased in maternal blood before the onset of pre‐eclampsia 18, 19. Changes in placental release are thought to underlie these alterations, as immunostaining for sFlt‐1 is increased, and for PlGF decreased, in pre‐eclamptic placentae compared to normal controls 20. Similarly, sFlt‐1 is increased and PlGF decreased in cultured trophoblast cells or placental villous explants exposed to oxidative stress 21. The pro‐angiogenic factors VEGF‐A and PlGF promote the survival and proliferation of endothelial cells and induce vascular permeability 22, 23. The binding of sFlt1 to free VEGF‐A and PlGF in the circulation reduces their availability and can act as a dominant‐negative receptor, thereby causing endothelial cell dysfunction, leading to hypertension, proteinuria and the other maternal systemic symptoms of pre‐eclampsia 24, 25. As a result, measurement of maternal circulating PlGF early in pregnancy has been used clinically to screen for both early‐onset pre‐eclampsia and FGR 17.

The level of PlGF transcripts is regulated by transcription factors, including metal responsive transcription factor 1 (MTF‐1), nuclear factor‐κB (NF‐κB) 26, 27, 28, as well as microRNA‐125b in hepatocellular cancer 29. In addition, in placental tissues the transcription factor glial cell missing 1 (GCM1) and the protein kinase A (PKA) signalling pathway up‐regulate its expression 28. The latter acts through cAMP‐responsive element binding proteins (CREBs) 30. However, the molecular mechanisms leading to down‐regulation of PlGF in the pathogenesis of pre‐eclampsia and FGR remain elusive.

Placental endoplasmic reticulum (ER) stress has recently been recognized as playing a central role in the pathophysiology of early‐onset pre‐eclampsia and FGR, but not in late‐onset pre‐eclampsia 31, 32. The ER is the major organelle for the biosynthesis of polypeptide hormones, growth factors and plasma membrane proteins, and initiates their folding and post‐translational modifications. Overloading of the ER with nascent proteins, or perturbation of its ionic homeostasis through a variety of pathological stimuli, can lead to an imbalance between the protein load and the capacity of the folding machinery. As a result, unfolded or misfolded proteins accumulate in the lumen, a condition known as ER stress 36. This leads to activation of signalling pathways, collectively known as the unfolded protein response (UPR). Initially, the UPR aims to restore normal ER function but, if the attempt fails, apoptotic cascades are activated to eliminate damaged cells. The UPR comprises three highly conserved signalling pathways, including PERK–eIF2α–ATF4, which attenuates non‐essential protein synthesis and increases antioxidant defence systems; ATF6, which up‐regulates ER chaperones (GRP78 and GRP94) to increase folding capacity; and IRE1–XBP1, which increases phospholipid biosynthesis and promotes misfolded protein degradation 33. These pathways are activated sequentially in a severity‐dependent manner 31, 34.

In addition, activation of the transcription factors in the UPR pathways regulates expression of genes involved in a wide range of cellular functions, such as redox processes, amino acid metabolism and angiogenesis 35, 36, 37, 38, 39. In this study, we elucidated a novel mechanism by which ER stress potentially modulates maternal endothelial cell activation via down‐regulation of PlGF gene expression in early‐onset pre‐eclampsia.

## Materials and methods

### Study population and placental sample collection

All placental samples were obtained with local ethical permission and the patients' informed written consent. The detailed criteria for recruitment of patients for this study have been described previously 40. Briefly, the control group was from healthy normotensive term patients who displayed no abnormalities on routine scans, while the pre‐eclamptic group was from patients with new‐onset hypertension (≥140/90 mmHg) observed on at least two separate occasions, 6 h or more apart, combined with proteinuria (a 24 h urine sample showing ≥ 300 mg/24 h). Women with pre‐pregnancy diseases such as hypertension, diabetes mellitus or pre‐existing renal disease were excluded. All placentae were obtained from elective, non‐laboured caesarean deliveries. For each placenta, four to six small pieces of tissue from separate lobules were rinsed three times in saline, blotted and snap‐frozen in liquid nitrogen within 10 min of delivery; the samples were stored at −80 °C. The clinical features of the pregnancies were described previously 32.

### Cell culture

Human choriocarcinoma JEG‐3 and BeWo cells were cultured as described previously 34, 41.

### Western blot analysis

Western blots were performed on both cell lysates and culture media to quantify relative total levels or phosphorylated levels of specific proteins. The details of antibodies and procedures are described in Supplementary materials and methods (see supporting information) and in a previous study 34.

### In vitro hypoxia–reoxygenation experiments

For hypoxia–reoxygenation (H/R) challenge, cells were cultured in normal growth medium without serum in an incubator that allowed precise and variable control of oxygen (ExVivo, BioSpherix, Lacona, NY, USA) and the experiments were performed as described in a previous study 32.

### Quantitative real‐time RT–PCR analysis

Total RNA was isolated using RNeasy Mini Kits (Qiagen, Manchester, UK), according to the manufacturer's instructions, and the quantitative PCR performed using SYBR Green JumpStart kits (Sigma, Dorset, UK). Details of the primer sequences (Table S1) and other procedures are described in Supplementary materials and methods (see supporting information). The PlGF primers detected three of the four isoforms of PlGF, including PlGF‐1, −2 and −3.

### Small RNA interference

siRNA‐mediated knockdown of transcripts of interest in BeWo cells was performed using Lipofectamine RNAiMAX transfection reagent (Life Technologies, Paisley, UK) for 48 h, according to the manufacturer's instructions. Details of siRNAs and the experimental procedures are described in Supplementary materials and methods (see supporting information)

### Immunohistochemistry

Immunohistochemistry was performed as previously described 42. The details of antibodies and specific conditions for each antibody are described in Supplementary materials and methods (see supporting information).

### Statistical analysis

Differences were tested using non‐parametric Kruskal–Wallis test with Dunn's multiple comparison test. Correlations between PlGF mRNA and ATF4 or ATF6α or ATF6β mRNAs were tested using Pearson's correlation. Power regression lines were fitted to display relationships. For cell culture experiments, both the Mann–Whitney test and two‐tailed Student's t‐test were used as appropriate. All statistical analyses were performed using GraphPad Prism v. 6.0, with p ≤ 0.05 considered significant.

## Results

### Severity of ER stress is negatively correlated with PlGF transcript and protein levels in pathological placentae

**Figure 1:**
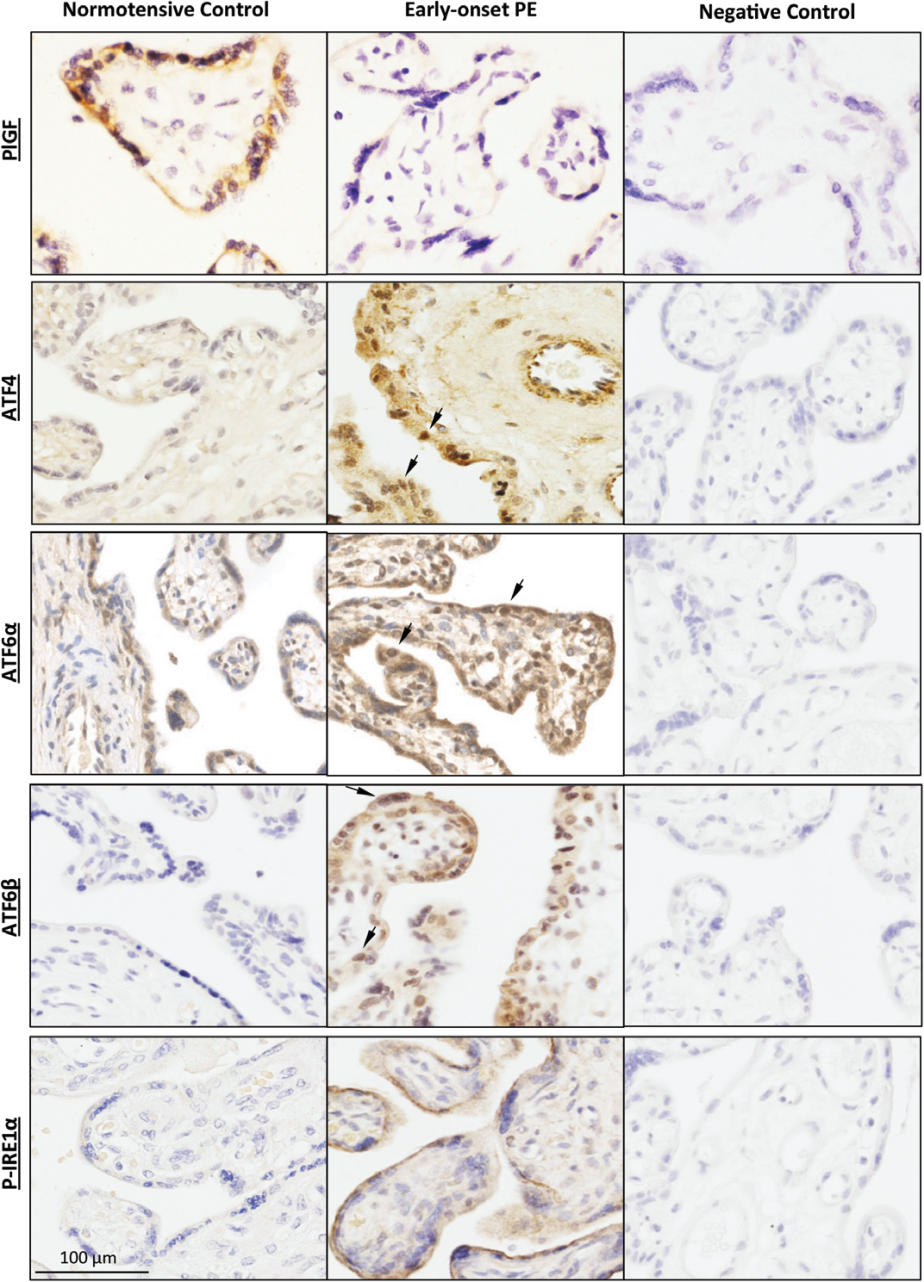
Placental ER stress is associated with lower PlGF expression in pre‐eclamptic placentae. Immunohistochemical staining showed the expression and localization of PlGF, ATF4, ATF6α, ATF6β and P‐IRE1α in both pre‐eclamptic and normotensive control placental sections using corresponding specific primary antibodies. Negative controls were performed by omitting the primary antibodies; scale bar = 100 µm; arrows, nuclear localization of proteins

Oxidative stress is a strong inducer of ER stress 34 and lowers PlGF in the placenta 21. We therefore investigated the potential role of ER stress in the regulation of PlGF synthesis. In the placenta, PlGF is predominately localized in the syncytiotrophoblast, and its level is reduced in placentae from cases of early‐onset pre‐eclampsia (Figure 1). We first examined activation of the three UPR pathways using immunohistochemistry. Interestingly, the activity of PERK (which is indicated by ATF4), ATF6 (which has two isoforms, α and β), and IRE1α were all increased, and located mainly in the syncytiotrophoblast and fetal endothelial cells (Figure 1). ATF4, ATF6α and ATFβ are transcription factors, and their activation promotes nuclear translocation. Indeed, increased ATF4 and ATF6α and ATFβ immunoreactivity was found mainly in the syncytiotrophoblastic nuclei, implicating them in transcriptional regulation in the pathological placentae.

**Figure 2:**
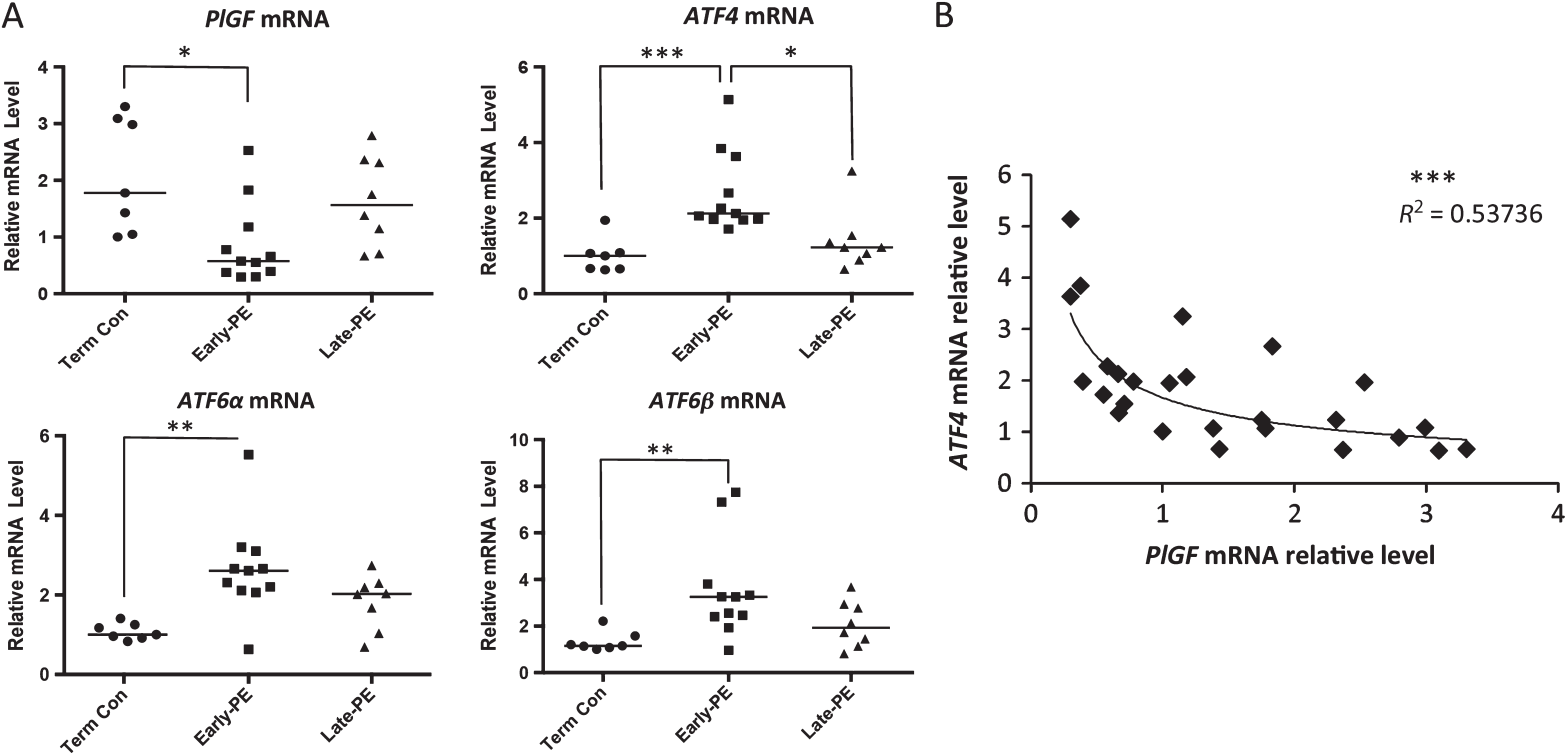
Placental ER stress is associated with lower PlGF mRNA levels in pre‐eclamptic placentae. (A) Quantitative real‐time RT–PCR was used to measure transcript levels of PlGF, ATF4, ATF6α and ATF6β in early‐PE, late‐PE and NTC placentae; data are presented as Dot‐plots with median; the differences among early‐PEs, late‐PEs and NTCs was analysed by a non‐parametric Kruskal–Wallis test with Dunn's multiple comparison test; early‐PE, n = 11; late‐PE, n = 8; and NTC, n = 7; *p ≤ 0.05, **p ≤ 0.01, ***p ≤ 0.001. (B) The correlation between ATF4 and PlGF mRNA levels was plotted for all placental samples; a power regression line was fitted to display the relationship between the two transcripts; ***p ≤ 0.001

We next investigated whether the low level of PlGF in placentae from pre‐eclamptic patients was regulated at the transcription level. Therefore, *PlGF* mRNA was measured in placentae from normotensive term controls (*n =* 7), early‐onset pre‐eclampsia (*n =* 10) and late‐onset pre‐eclampsia (*n =* 8). A significant reduction of *PlGF* mRNA was observed in placentae from early‐onset pre‐eclampsia, but not in those from late‐onset pre‐eclampsia, where levels were indistinguishable from the term controls (Figure 2A). Analysis of *ATF4*, *ATF6α* and *ATF6β* transcripts revealed that all three were similarly significantly elevated in placentae from early‐onset pre‐eclampsia, but not in those from the late‐onset cases (Figure 2A).

To test for any relationship between these ER stress‐mediated transcription factors and the *PlGF* mRNA level, correlations were plotted for *PlGF* mRNA against the transcript levels of *ATF4*, *ATF6α* and *ATF6β*. We observed a significant relationship (*p <* 0.001) between *ATF4* and *PlGF* transcripts, with the correlation coefficient reaching −0.733 (*R* ^2^ = 0.537) (Figure 2B). By contrast, no significant relationship was observed for *ATF6α* and *ATF6β* mRNA (see supplementary material, Figure S1). This result strongly suggests a potential role of ER stress in the negative regulation of *PlGF* transcription mediated by ATF4. Therefore, cell culture models and ER stress inducers were used to investigate the potential mechanism further.

### ER stress induces a severity‐dependent reduction in PlGF secretion and gene expression in trophoblast‐like cell lines

**Figure 3:**
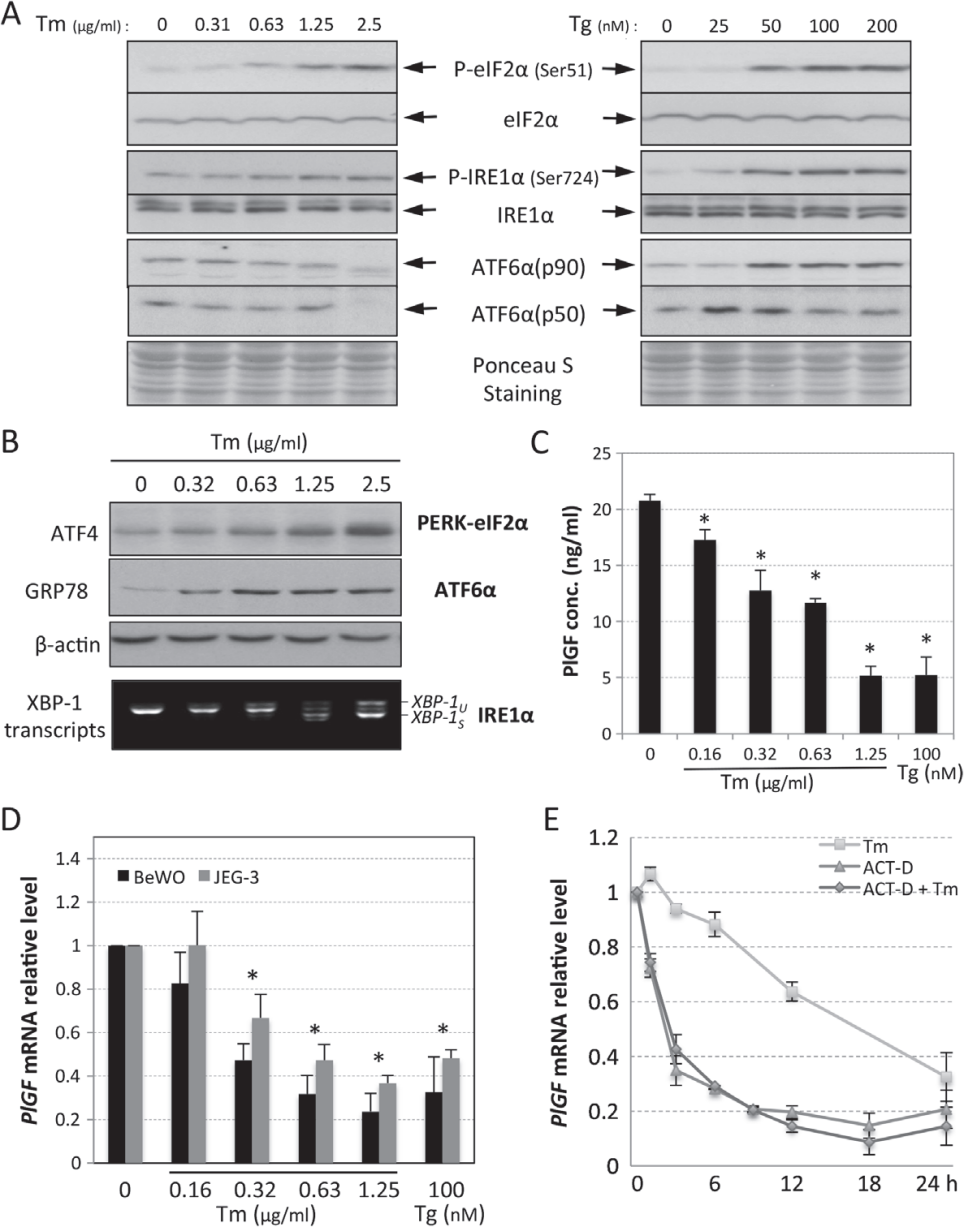
ER stress induces a severity‐dependent reduction of PlGF protein and mRNA in trophoblastic‐like cells. (A) Dose–response study of ER stress inducers, tunicamycin (Tm) and thapsigargin (Tg) in BeWo cells: the severity of ER stress, which is indicated by the degree of activation in three highly conserved UPR pathways PERK, IRE1 and ATF6, was measured by western blotting analysis, with primary antibodies specific to phosphorylated or total protein levels of P‐eIF2α (Ser51), eIF2α, P‐IRE1α (Ser724), IRE1α, ATF6α (p90) and ATF6α (p50); Ponceau S staining was used to show equal protein loading of the samples. (B) Activation of three UPR pathways in the tunicamycin dose–response study was indicated by their downstream effectors: ATF4 for PERK; GRP78 for ATF6 and XBP‐1 mRNA splicing for IRE1; western blotting was used to measure protein levels of ATF4 and GRP78 and β‐actin was used as the loading control, while RT–PCR was used to analyse mRNA splicing of XBP‐1. (C, D) ER stress severity‐dependent down‐regulates PlGF release and gene expression in trophoblast‐like cells. (C) ELISA was used to quantify the amount of PlGF released by BeWo cells after treating with various concentrations of ER stress inducers for 24 h; data are presented as mean ± SEM, n = 4. (D) Quantitative RT–PCR was used to measure PlGF mRNA levels in both BeWo and JEG‐3 cells after treatment with tunicamycin or thapsigargin for 24 h; data are presented as mean ± SEM, n = 4/group; *p < 0.05 compared to control. (E) Decrease of PlGF mRNA under ER stress is not the result of increased RNA degradation: PlGF mRNA stability was analysed using the global transcriptional inhibitor actinomycin D (1 µg/ml) in the presence or absence of Tm (0.625 µg/ml); qRT–PCR was used to measure PlGF mRNA level in a time‐course study, in which samples were collected after 0, 1, 3, 6, 9, 12, 18 and 24 h; data are presented as mean ± SEM, n = 3/time point/group

In order to eliminate cell type‐specific and drug‐specific effects, two human choriocarcinoma cell lines, JEG‐3 and BeWo, and two ER stress inducers, tunicamycin (Tm, an N‐linked glycosylation inhibitor) and thapsigargin (Tg, an inhibitor of sarco‐endoplasmic reticulum Ca^2+^ ATPase), were used for studies *in vitro*. We have previously reported a dose–response effect of tunicamycin on the activation of ER stress pathways and apoptosis in JEG‐3 cells 31. Therefore, the results presented in Figure 3A are from BeWo cells. Doses of tunicamycin of up to 0.63 µg/ml cause minimal cell death (3.2%), whereas 1.25 µg/ml and 2.5 µg/ml cause 40% and 56%, respectively 34. We performed a similar dose–response study for thapsigargin, and found an equivalent effect in induction of apoptosis. Doses < 100 nm caused minimal cell death after 24 h, and so this was the maximum concentration used for subsequent experiments. Increasing concentrations of thapsigargin induced dose‐dependent activation of all three UPR pathways, while with tunicamycin the PERK‐eIF2α and IRE1α arms were clearly activated, but not the ATF6α pathway. However, ATF6α is a glycoprotein containing three N‐glycosylation sites at the C‐terminus, and under‐glycosylation has been shown to increase its transcriptional activity 43. In Figure 3A, a gradual reduction in ATF6α electrophoretic mobility was observed as the concentration of tunicamycin increased, indicating a loss of N‐glycosylation and thereby an elevation of its transcriptional activity. Both ATF6α(p50) and ATF6α(p90) undergo proteosomal‐mediated degradation upon activation 44, 45. This might explain why ATF6α protein was greatly reduced at the lethal dosage of tunicamycin (2.5 µg/ml).

In order to reveal the activity of these pathways in response to tunicamycin, the downstream effectors of the PERK–eIF2α, ATF6α and IRE1α pathways, viz. ATF4, GRP78 and XBP‐1, respectively, were examined. Under ER stress, phosphorylation of eIF2α mediates an increase in transcription and translation of ATF4 46; activation of ATF6α up‐regulates *GRP78* gene expression 47, while the increased endoribonuclease activity of activated IRE1α facilitates *XBP‐1* mRNA splicing 48. As can be seen in Figure 3B, there was a dose‐dependent increase of ATF4, GRP78 and splicing of *XBP‐1* mRNA, confirming activation of all three UPR pathways in response to tunicamycin. Crucially, increasing severity of ER stress was closely associated with decreased secretion of PlGF by the cells (Figure 3C).

The reduced levels of *PlGF* mRNA measured in placentae from early‐onset pre‐eclampsia in Figure 2A suggest that the decrease in maternal blood concentrations PlGF seen in pre‐eclampsia are likely to result from the reduction of *PlGF* mRNA. We therefore examined *PlGF* transcripts under ER stress in both BeWo and JEG‐3 cells. Indeed, *PlGF* mRNA was reduced in an ER stress severity‐dependent manner in both cell types (Figure 3D), demonstrating a similar profile to the secreted PlGF (Figure 3C). Both transcription and RNA degradation contribute to the transcript level. *PlGF* mRNA stability was investigated using the global transcription inhibitor, actinomycin D (Act‐D). We treated cells in the presence or absence of tunicamycin and followed the degradation of the *PlGF* transcripts over time. As shown in Figure 3E, *PlGF* mRNA levels under ER stress were unchanged in the absence of transcription. This indicates that the reduction was not due to increased mRNA degradation and hence the observed changes in *PlGF* transcript level were due to changes in transcription. Furthermore, comparison of the rates of *PlGF* mRNA decline induced by tunicamycin and Act‐D alone shows that the rate with tunicamycin was relatively linear, whereas with Act‐D there was a 60% decrease in the first 3 h.

### Transcription of PlGF is negatively regulated by ATF4 and ATF6β

**Figure 4:**
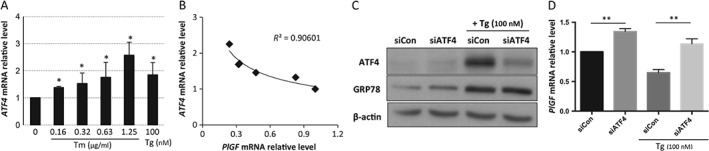
Expression of PlGF mRNA is negatively regulated by ATF4 in a trophoblastic‐like cell. (A) Dose‐dependent increase of ATF4 mRNA under ER stress: BeWo cells were cultured in normal growth medium without serum in various concentrations of the ER stress inducers tunicamycin (Tm) and thapsigargin (Tg) for 24 h; normalized ATF4 mRNA level was measured by qPCR; data are presented as mean ± SEM, n = 3; *p < 0.05 compared to control. (B) Negative correlation between PlGF and ATF4 mRNA relative levels in BeWo cells in response to ER stress; the correlation was plotted using the data in Figures 4A and 3D. (C) siATF4 greatly reduces ATF4 protein level in both control and treated conditions: cell lysates were isolated and subjected to western blotting analysis with ATF4‐specific antibody; GRP78 level was used to indicate existence of ER stress upon thapsigargin treatment and β‐actin was used as the protein loading control. (D) Suppression of ATF4 increases PlGF mRNA: BeWo cells were transfected with non‐targeting control small interfering RNAs (siCon) or ATF4 siRNA (siAtf4); after 48 h of transfection, the cells were treated with thapsigargin for 24 h; PlGF and ATF4 mRNA levels were measured by quantitative RT–PCR normalized to internal controls, TBP and GAPDH; data are presented as mean ± SEM; n = 8; **p < 0.01

The results presented in Figure 2B indicate a strong negative correlation between the levels of the transcription factor *ATF4* and *PlGF* mRNA in placentae from pre‐eclampsia. Therefore, we examined whether the same correlation existed in the cell culture model in response to ER stress. There was a dose‐dependent increase of *ATF4* mRNA upon increasing concentrations of tunicamycin (Figure 4A), which correlated closely (*R* ^2^ = 0.91) with the decline in *PlGF* mRNA (Figure 4B).

To provide direct evidence of a role of ATF4 in the regulation of *PlGF* transcription, we specifically knocked down *ATF4* transcripts using siRNA. Application of *siATF4* reduced *ATF4* mRNA and protein by > 70% in both untreated and thapsigargin‐treated conditions (Figure 4C; see supplementary material, Figure S2A). *siATF4* treatment also up‐regulated *PlGF* mRNA by 34% in untreated cells (Figure 4D). By contrast, in the presence of thapsigargin, *PlGF* transcripts were suppressed by 35% in *siCon* cells, but were restored by over 75% in *siATF4*‐treated cells (Figure 4D), confirming the role of ATF4 in the negative regulation of *PlGF* transcription.

**Figure 5:**
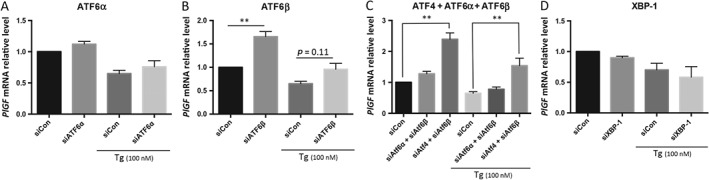
ATF6β acts synergistically with ATF4 in down‐regulation of PlGF transcription, while ATF6α and XBP‐1 are not involved in the regulation: siRNAs specific for siATF4, ATF6α, ATF6β and siXBP‐1 were used individually or in a combination to knock down corresponding gene(s) for 48 h before treating with thapsigargin for an additional 24 h in BeWo cells; PlGF mRNA levels were measured by qPCR normalized to internal controls, TBP and GAPDH; data are presented as mean ± SEM; n = 4–8; **p < 0.01. (A) ATF6α; (B) ATF6β; (C) ATF4–ATF6α–ATF6β; (D) XBP‐1

In addition to ATF4, the transcription factors ATF6α, ATF6β and XBP‐1 are also involved in UPR pathways in response to ER stress. Nuclear localization of ATF6α and ATF6β in placentae from early‐onset pre‐eclampsia (Figure 1) was increased and our previous studies showed increased *spliced XBP‐1* (activated form) mRNA in similar placentae 31. We therefore knocked down *ATF6α*, *ATF6β* and *XBP‐1* transcripts using specific siRNAs. *ATF6α*, *ATF6β* and *XBP‐1* mRNAs were suppressed by > 70% (see supplementary material, Figure S2B, C). *siATF6α* treatment did not affect *ATF6β* expression and vice versa (see supplementary material, Figure S2B). Levels of the *PlGF* transcript were not affected by *siATF6α* and *siXBP‐1* in both untreated and thapsigargin‐treated cells (Figure 5A, D). However, following *siATF6β* treatment, we observed a 65% increase of *PlGF* mRNA in untreated cells, although the increase did not reach statistical significance (*p =* 0.11) in the thapsigargin‐treated condition (Figure 5B).

Activated ATF6β has been shown to interact with ATF6α, thereby inhibiting UPR‐mediated gene expression 49. Therefore, we investigated whether ATF6β might modulate ATF6α‐ or ATF4‐mediated *PlGF* gene expression. siRNA‐mediated double knockdown of *ATF6β + ATF4* and *ATF6α + β* genes was performed in the presence of thapsigargin. Application of *siATF6α* + *β* reduced *ATF6α* and *ATF6β* transcript levels by 80% and 60%, respectively (see supplementary material, Figure S2B). Knockdown of both *ATF6α* and *β* did not significantly enhance *PlGF* mRNA in either untreated or thapsigargin‐treated cells (Figure 5C). By contrast, in the *siATF6β + siATF4* double knockdown, we observed a synergistic effect in up‐regulation of *PlGF* transcript levels. In comparison to *siCon*, *siATF6β + siATF4* caused a 2.4‐fold increase of *PlGF* mRNA in both untreated and thapsigargin‐treated cells (Figure 5C).

### Hypoxia–reoxygenation modulates PlGF transcription inhibition, which is partially regulated through ATF4 and ATF6β

**Figure 6:**
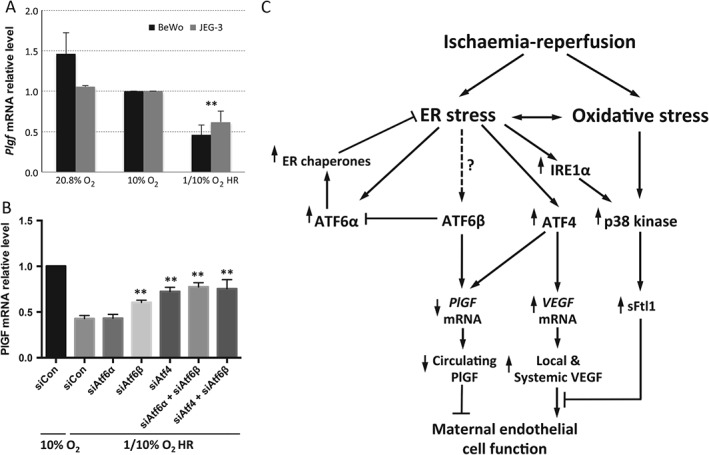
Expression of PlGF is partially regulated by ATF4 and ATF6β in response to hypoxia–reoxygenation. (A) Hypoxia–reoxygenation (H/R) suppresses PlGF mRNA: BeWo and JEG‐3 cells were cultured under serum‐free conditions and subjected to repetitive H/R in a 6 h cyclic pattern between 1% O_2_ and 10% O_2_ for 24 h; the control cells were incubated at both 20% and 10% O_2_; PlGF mRNA relative levels were measured by qPCR. (B) ATF4 and ATF6β, but not ATF6α, partially regulates PlGF mRNA level: BeWo cells were transfected with siATF4, siATF6α, siATF6β or, in combination siATF4 + ATF6β and siATF6α + siATF6β, while non‐targeting gene control (siCon) for 48 h prior to being subjected to H/R conditions for 24 h; relative levels of PlGF mRNA were analysed by qPCR; data are expressed as mean ± SEM; n = 4; **p < 0.01. (C) Flow chart showing the potential mechanisms by which ischaemia–reperfusion‐induced placental ER and oxidative stress may modulate maternal endothelial cell function through regulation of PlGF, VEGF‐A and sFlt1 levels

Poor placentation induces hypoxia–reperfusion, resulting in placental oxidative stress, a key feature of the pathophysiology of growth restriction and early‐onset pre‐eclampsia 50. Therefore, an *in vitro* model of repetitive hypoxia–reoxygenation (rHR), in which cells were repeatedly exposed to 6 h cycles of 1% and 10% O_2_ for 24 h, was used as a more physiological model of stress. *PlGF* mRNA was reduced ∼50% after 24 h of rH/R (Figure 6A). Application of *siATF4* and *siATF6β*, individually or in combination, partially restored *PlGF* mRNA levels but was not as efficient as when used with the ER stress‐inducer thapsigargin (Figure 6B). These results suggest that other transcription factors, such as MTF1 and NF‐κB, may contribute to regulation of *PlGF* transcription under oxidative stress.

## Discussion

Maternal circulating PlGF increases as pregnancy advances, reaching a peak at around 29–32 weeks before declining thereafter 18. In pregnancies complicated with early‐onset pre‐eclampsia and growth restriction, circulating PlGF concentrations are lower throughout gestation compared to normal controls 18, 51, 52. The reduction of circulating PlGF is much more pronounced compared to age‐matched controls in early‐onset pre‐eclampsia than in the late‐onset form of the syndrome 53. Ranking the three pathologies according to their circulating PlGF levels reveals that early‐onset pre‐eclampsia has the lowest concentration, followed by growth restriction and late‐onset pre‐eclampsia. These clinical observations suggest a negative relationship between the severity of placental ER stress and the maternal circulating PlGF level. In this study, we demonstrate that PlGF is indeed regulated at the transcriptional level by the UPR transcription factors ATF4 and ATF6β, resulting in a reduction of PlGF secretion.

A limitation of our study is that we were not able to compare the pathological placentae with age‐matched controls. Most pre‐term deliveries of normotensive pregnancies occur by vaginal delivery, due to conditions such as chorioamnionitis. These placentae display high levels of ER stress, due to either the underlying pathology or the delivery process 35, and so are not suitable as controls in this setting. Obtaining normal placentae delivered by caesarean section at 25 weeks, the earliest pathological cases studied here, is almost impossible ethically. Nonetheless, we do not consider that our findings can be attributed to differences in gestational age. First, concentrations of PlGF fall in late gestation, as described above, and so would be expected to be lower, rather than higher, in the term controls. Second, the strong negative correlation between between *PlGF* and *ATF4* mRNAs shown in Figure 2B is independent of gestational age and placental pathology.

Due to the unique role of PlGF in the modulation of VEGF‐A signalling during endothelial cell homeostasis and angiogenesis, especially in a stressed environment 54, the low circulating PlGF concentration is widely accepted as one of the key contributors to maternal endothelial cell dysfunction in pre‐eclampsia 3, 20, 51. Surprisingly, another key angiogenic factor, VEGF‐A, is increased in the circulation of pre‐eclamptic patients 55. This may also reflect placental ER stress, since ATF4 and IRE1α positively regulate *VEGF* gene expression 35, 56. However, the biological activity of VEGF‐A is complicated by the fact that it is largely bound to its antagonist sFlt1, which is also elevated. Consequently, there is a reduction in free circulating VEGF‐A, further compromising maternal vascular function 24. Placental oxidative stress induces secretion of sFlt1 through p38 kinase signalling 57. ER stress also activates p38 kinase via the IRE1α–TRAF2 pathway 58, 59, thereby potentially contributing to modulation of sFlt1 secretion.

All three UPR pathways are strongly activated in placentae from early‐onset pre‐eclampsia, but to only subtle degrees in late‐onset cases 32. Our results are thus consistent with the remarkable increase of maternal circulating sFlt1 and reduction of PlGF in early‐onset pre‐eclampsia, and the minimal changes seen in the late‐onset form of the syndrome 53. Taken together, these findings suggest a potential vital role of placental ER stress in the modulation of maternal endothelial cell function via regulation of the synthesis and secretion of pro‐angiogenic and anti‐angiogenic factors in response to haemodynamic challenges (Figure 6).

The exact mechanism of the synergistic inhibitory effect of ATF4 and ATF6β in the regulation of PlGF transcription remains to be elucidated. However, a recent study reported that there are two functional cAMP responsive elements (CREs) in the *PlGF* promoter, and that the CREBs can up‐regulate *PlGF* gene expression upon cAMP stimulation 30. Coincidently, ATF4 and ATF6β are also known as cAMP‐responsive element binding protein 2 (CREBP2) and cAMP‐responsive element‐binding protein‐like 1 (CREBPL‐1), respectively. They belong to a family of DNA‐binding proteins including the AP‐1 family of transcription factors, cAMP‐response element binding proteins (CREBs) and CREB‐like proteins, indicating their potential interaction with CREs. Although both ATF6α and ATF6β show high structural similarity to each other, undergo the same proteolytic cleavage to generate their activated forms and bind to the same regulatory elements, they exhibit isoform‐specific transcriptional activation and stability characteristics 60, 61. Activated ATF6β is a relatively poor inducer of UPR response genes, but has much greater stability than activated ATF6α 60, 61. An *in vitro* DNA‐binding experiment demonstrated that recombinant activated ATF6β inhibits the binding of recombinant activated ATF6α to an UPR response element from the target gene promoter 49. We therefore speculate that one of the potential mechanisms is ATF4 and ATF6β act synergistically to interact directly or indirectly with CRE in the *PlGF* promoter, thereby inhibiting CREBs‐mediated *PlGF* transcription. This hypothesis is supported by the observation that the increase of both placental and maternal circulating adrenomedullin, a ligand that increases intracellular cAMP via G‐protein‐coupled receptors and adenylate cyclase, in pre‐eclampsia does not result in the expected rise in PlGF 62, 63.

The majority of secreted proteins are glycoproteins, and glycosylation is crucial for their stability, functional activity and half‐life in the circulation 64. Our previous publication demonstrated that glycosylation of secreted proteins is altered upon ER stress, and that their activity was compromised 65. PlGF contains two glycosylation sites, and so its activity is expected to be influenced by ER stress. Therefore, we speculate that ER stress not only suppresses *PlGF* transcription but may also modulate its bioactivity. Further experiments will be required to address this hypothesis. Furthermore, ER stress could also affect the fetal vasculature of the placenta through modulation of the expression and activities of the PlGF and VEGF receptors, VEGFR1 and VEGFR2/KDR. In placentae from early‐onset pre‐eclampsia, immunostaining of ATF4 and ATF6α was also increased in the endothelial cells of the fetal capillaries, indicating the existence of ER stress in these vessels (Figure 1). VEGFR1 and VEGFR2 are present in both the syncytiotrophoblast and the capillary endothelial cells 66, and VEGFR2 expression is decreased in pre‐eclamptic placentae 67. A recent study reported that administration of an ER stress inducer (tunicamycin) to pregnant mice is associated with a reduction of *VEGFR1* and *VEGFR2* mRNA expression in the placenta 68, suggesting a potential role for ER stress in regulation of these receptors. Additionally, it has been demonstrated that the glycosylation of VEGFR2 is crucial for its activity 69, and likely to be influenced by ER stress. Indeed, in placentae from pre‐eclampsia, VEGFR1 (Flt1) and endoglin are hyperglycosylated compared to normotensive control placentae 20.

To conclude, our previous publications demonstrated that in cases of early‐onset pre‐eclampsia and FGR placental ER stress causes protein synthesis inhibition that contributes to the growth‐restricted phenotype. In this study, we reveal that another consequence of placental ER stress is the down‐regulation of *PlGF* transcription, which is likely to contribute to the reduction in maternal circulating PlGF, which in turn may modulate maternal endothelial cell function and thereby contribute to the pathophysiology of the syndrome. These new findings further support the concept that alleviation of placental ER stress may provide a new therapeutic target for the treatment of early‐onset pre‐eclampsia and FGR.

## Author contributions

MM, GJB, DSC‐J and HWY designed the study; MO identified and obtained consent from the patients and obtained the placental samples; MM, TC‐D and HWY performed the experiments; and MM, GJB, DSC‐J and HWY analysed the data and wrote the paper. All authors approved the final version.

**SUPPLEMENTARY MATERIAL ON THE INTERNET**

The following supplementary material may be found in the online version of this article:

Supplementary materials and methods

**Figure S1.** No correlation was found between PlGF and ATF6α or ATF6β in pre‐eclamptic placentae

**Figure S2.** Small RNA interference treatment is effective in knocking down of corresponding transcripts

**Table S1.** The primer sequences used for quantitative real‐time RT‐PCR and their corresponding amplicon sizes.

## Supporting information


**Appendix S1**. Supporting information

**Figure S1** No correlation was found between PlGF and ATF6α or ATF6β in pre‐eclamptic placentae. qPCR was used to measure transcript levels of PlGF, ATF6α and ATF6β in early‐PE, late‐PE and NTC placentae; a correlation curve was plotted for all placental samples between ATF6α and PlGF or ATF6β and PlGF mRNA levels, using data from Figure 1B. A power regression line was fitted to display the relationship between genes. Pearson correlation was performed, with p ≤ 0.05 considered significant

**Figure S2** Small RNA interference treatment is effective in knocking down corresponding transcripts. BeWo cells (∼40% confluent) were used for transfection of corresponding gene‐specific siRNA, using the Lipofectamine RNAiMAX transfection reagent for 48 h before the ER stress treatment for an additional 24 h. (A, C, D) qPCR was used to quantify the relevant transcript and GAPDH and TBP were used as internal controls: (A) ATF4; (B) ATF6α and ATF6β; (C) XBP‐1; data are presented as mean ± SEM, n = 3; for ATF6α and ATF6β, only a single experiment was performed; **p < 0.01

**Table S1** The primer sequences used for quantitative real‐time RT‐PCR and their corresponding amplicon sizes.

## References

[1] Khan KS , Wojdyla D , Say L , *et al.* WHO analysis of causes of maternal death: a systematic review. Lancet 2006; 367: 1066–1074. 16581405 10.1016/S0140-6736(06)68397-9

[2] Roberts JM , Bell MJ . If we know so much about preeclampsia, why haven't we cured the disease? J Reprod Immunol 2013; 99: 1–9. 23890710 10.1016/j.jri.2013.05.003 PMC4066309

[3] Lapaire O , Shennan A , Stepan H . The preeclampsia biomarkers soluble fms‐like tyrosine kinase‐1 and placental growth factor: current knowledge, clinical implications and future application. Eur J Obstet Gynecol Reprod Biol 2010; 151: 122–129. 20457483 10.1016/j.ejogrb.2010.04.009

[4] Redman CW , Sargent IL . Latest advances in understanding preeclampsia. Science 2005; 308: 1592–1594. 15947178 10.1126/science.1111726

[5] Tranquilli AL , Brown MA , Zeeman GG , *et al.* The definition of severe and early‐onset preeclampsia. Statements from the International Society for the Study of Hypertension in Pregnancy (ISSHP). Pregnancy Hypertens 2013; 3: 44–47. 26105740 10.1016/j.preghy.2012.11.001

[6] Roberts JM , Redman CW . Pre‐eclampsia: more than pregnancy‐induced hypertension. Lancet 1993; 341: 1447–1451. 8099148 10.1016/0140-6736(93)90889-o

[7] Powe CE , Levine RJ , Karumanchi SA . Preeclampsia, a disease of the maternal endothelium: the role of antiangiogenic factors and implications for later cardiovascular disease. Circulation 2011; 123: 2856–2869. 21690502 10.1161/CIRCULATIONAHA.109.853127 PMC3148781

[8] Verlohren S , Stepan H , Dechend R . Angiogenic growth factors in the diagnosis and prediction of pre‐eclampsia. Clin Sci (Lond) 2012; 122: 43–52. 21929511 10.1042/CS20110097

[9] Maglione D , Guerriero V , Viglietto G , *et al.* Isolation of a human placenta cDNA coding for a protein related to the vascular permeability factor. Proc Natl Acad Sci USA 1991; 88: 9267–9271. 1924389 10.1073/pnas.88.20.9267 PMC52695

[10] Autiero M , Waltenberger J , Communi D , *et al.* Role of PlGF in the intra‐ and intermolecular crosstalk between the VEGF receptors Flt1 and Flk1. Nat Med 2003; 9: 936–943. 12796773 10.1038/nm884

[11] Hauser S , Weich HA . A heparin‐binding form of placenta growth factor (PlGF‐2) is expressed in human umbilical vein endothelial cells and in placenta. Growth Factors 1993; 9: 259–268. 8148155 10.3109/08977199308991586

[12] Khaliq A , Li XF , Shams M , *et al.* Localisation of placenta growth factor (PIGF) in human term placenta. Growth Factors 1996; 13: 243–250; colour plates I, II, pre bk cov. 8919031 10.3109/08977199609003225

[13] Cao Y , Ji WR , Qi P , *et al.* Placenta growth factor: identification and characterization of a novel isoform generated by RNA alternative splicing. Biochem Biophys Res Commun 1997; 235: 493–498. 9207183 10.1006/bbrc.1997.6813

[14] Khaliq A , Dunk C , Jiang J , *et al.* Hypoxia down‐regulates placenta growth factor, whereas fetal growth restriction up‐regulates placenta growth factor expression: molecular evidence for 'placental hyperoxia' in intrauterine growth restriction. Lab Invest 1999; 79: 151–170. 10068204

[15] Yang W , Ahn H , Hinrichs M , *et al.* Evidence of a novel isoform of placenta growth factor (PlGF‐4) expressed in human trophoblast and endothelial cells. J Reprod Immunol 2003; 60: 53–60. 14568677 10.1016/s0165-0378(03)00082-2

[16] Park JE , Chen HH , Winer J , *et al.* Placenta growth factor. Potentiation of vascular endothelial growth factor bioactivity, *in vitro* and *in vivo*, and high affinity binding to Flt‐1 but not to Flk‐1/KDR. J Biol Chem 1994; 269: 25646–25654. 7929268

[17] Cowans NJ , Stamatopoulou A , Matwejew E , *et al.* First‐trimester placental growth factor as a marker for hypertensive disorders and SGA. Prenat Diagn 2010; 30: 565–570. 20509158 10.1002/pd.2525

[18] Levine RJ , Maynard SE , Qian C , *et al.* Circulating angiogenic factors and the risk of preeclampsia. N Engl J Med 2004; 350: 672–683. 14764923 10.1056/NEJMoa031884

[19] Levine RJ , Lam C , Qian C , *et al.* Soluble endoglin and other circulating antiangiogenic factors in preeclampsia. N Engl J Med 2006; 355: 992–1005. 16957146 10.1056/NEJMoa055352

[20] Gu Y , Lewis DF , Wang Y . Placental productions and expressions of soluble endoglin, soluble fms‐like tyrosine kinase receptor‐1, and placental growth factor in normal and preeclamptic pregnancies. J Clin Endocrinol Metab 2008; 93: 260–266. 17956952 10.1210/jc.2007-1550 PMC2190747

[21] Cindrova‐Davies T , Yung HW , Johns J , *et al.* Oxidative stress, gene expression, and protein changes induced in the human placenta during labor. Am J Pathol 2007; 171: 1168–1179. 17823277 10.2353/ajpath.2007.070528 PMC1988867

[22] Keck PJ , Hauser SD , Krivi G , *et al.* Vascular permeability factor, an endothelial cell mitogen related to PDGF. Science 1989; 246: 1309–1312. 2479987 10.1126/science.2479987

[23] Leung DW , Cachianes G , Kuang WJ , *et al.* Vascular endothelial growth factor is a secreted angiogenic mitogen. Science 1989; 246: 1306–1309. 2479986 10.1126/science.2479986

[24] Maynard SE , Min JY , Merchan J , *et al.* Excess placental soluble fms‐like tyrosine kinase 1 (sFlt1) may contribute to endothelial dysfunction, hypertension, and proteinuria in preeclampsia. J Clin Invest 2003; 111: 649–658. 12618519 10.1172/JCI17189 PMC151901

[25] Cindrova‐Davies T , Sanders DA , Burton GJ , *et al.* Soluble FLT1 sensitizes endothelial cells to inflammatory cytokines by antagonizing VEGF receptor‐mediated signalling. Cardiovasc Res 2011; 89: 671–679. 21139021 10.1093/cvr/cvq346 PMC3028975

[26] Green CJ , Lichtlen P , Huynh NT , *et al.* Placenta growth factor gene expression is induced by hypoxia in fibroblasts: a central role for metal transcription factor‐1. Cancer Res 2001; 61: 2696–2703. 11289150

[27] Cramer M , Nagy I , Murphy BJ , *et al.* NF‐κB contributes to transcription of placenta growth factor and interacts with metal responsive transcription factor‐1 in hypoxic human cells. Biol Chem 2005; 386: 865–872. 16164411 10.1515/BC.2005.101

[28] Chang M , Mukherjea D , Gobble RM , *et al.* Glial cell missing 1 regulates placental growth factor (*PGF*) gene transcription in human trophoblast. Biol Reprod 2008; 78: 841–851. 18160678 10.1095/biolreprod.107.065599 PMC2442461

[29] Alpini G , Glaser SS , Zhang JP , *et al.* Regulation of placenta growth factor by microRNA‐125b in hepatocellular cancer. J Hepatol 2011; 55: 1339–1345. 21703189 10.1016/j.jhep.2011.04.015 PMC3184370

[30] Depoix C , Tee MK , Taylor RN . Molecular regulation of human placental growth factor (*PlGF*) gene expression in placental villi and trophoblast cells is mediated via the protein kinase a pathway. Reprod Sci 2011; 18: 219–228. 21135203 10.1177/1933719110389337 PMC3343062

[31] Yung HW , Calabrese S , Hynx D , *et al.* Evidence of placental translation inhibition and endoplasmic reticulum stress in the etiology of human intrauterine growth restriction. Am J Pathol 2008; 173: 451–462. 18583310 10.2353/ajpath.2008.071193 PMC2475782

[32] Yung HW , Atkinson D , Campion‐Smith T , *et al.* Differential activation of placental unfolded protein response pathways implies heterogeneity in causation of early‐ and late‐onset pre‐eclampsia. J Pathol 2014; 234: 262–276. 24931423 10.1002/path.4394 PMC4277692

[33] Ron D , Walter P . Signal integration in the endoplasmic reticulum unfolded protein response. Nat Rev Mol Cell Biol 2007; 8: 519–529. 17565364 10.1038/nrm2199

[34] Yung HW , Korolchuk S , Tolkovsky AM , *et al.* Endoplasmic reticulum stress exacerbates ischemia–reperfusion‐induced apoptosis through attenuation of Akt protein synthesis in human choriocarcinoma cells. FASEB J 2007; 21: 872–884. 17167073 10.1096/fj.06-6054com PMC1885550

[35] Ghosh R , Lipson KL , Sargent KE , *et al.* Transcriptional regulation of VEGF‐A by the unfolded protein response pathway. PLoS One 2010; 5: e9575. 20221394 10.1371/journal.pone.0009575 PMC2833197

[36] Harding HP , Zhang Y , Zeng H , *et al.* An integrated stress response regulates amino acid metabolism and resistance to oxidative stress. Mol Cell 2003; 11: 619–633. 12667446 10.1016/s1097-2765(03)00105-9

[37] Adachi Y , Yamamoto K , Okada T , *et al.* ATF6 is a transcription factor specializing in the regulation of quality control proteins in the endoplasmic reticulum. Cell Struct Funct 2008; 33: 75–89. 18360008 10.1247/csf.07044

[38] Lange PS , Chavez JC , Pinto JT , *et al.* *ATF4* is an oxidative stress‐inducible, prodeath transcription factor in neurons *in vitro* and *in vivo* . J Exp Med 2008; 205: 1227–1242. 18458112 10.1084/jem.20071460 PMC2373852

[39] Yamaguchi S , Ishihara H , Yamada T , *et al.* *ATF4*‐mediated induction of 4E‐BP1 contributes to pancreatic β cell survival under endoplasmic reticulum stress. Cell Metab 2008; 7: 269–276. 18316032 10.1016/j.cmet.2008.01.008

[40] Wikstrom AK , Nash P , Eriksson UJ , *et al.* Evidence of increased oxidative stress and a change in the plasminogen activator inhibitor (PAI)‐1:PAI‐2 ratio in early‐onset but not late‐onset preeclampsia. Am J Obstet Gynecol 2009; 201: 597 e591–598. 19683696 10.1016/j.ajog.2009.06.024

[41] Colleoni F , Padmanabhan N , Yung HW , *et al.* Suppression of mitochondrial electron transport chain function in the hypoxic human placenta: a role for miRNA‐210 and protein synthesis inhibition. PLoS One 2013; 8: e55194. 10.1371/journal.pone.0055194 PMC3559344 23383105

[42] Yung HW , Cox M , Tissot van Patot M , *et al.* Evidence of endoplasmic reticulum stress and protein synthesis inhibition in the placenta of non‐native women at high altitude. FASEB J 2012; 26: 1970–1981. 22267338 10.1096/fj.11-190082 PMC3336782

[43] Hong M , Luo S , Baumeister P , *et al.* Underglycosylation of ATF6 as a novel sensing mechanism for activation of the unfolded protein response. J Biol Chem 2004; 279: 11354–11363. 14699159 10.1074/jbc.M309804200

[44] Ye J , Rawson RB , Komuro R , *et al.* ER stress induces cleavage of membrane‐bound ATF6 by the same proteases that process SREBPs. Mol Cell 2000; 6: 1355–1364. 11163209 10.1016/s1097-2765(00)00133-7

[45] Hong M , Li M , Mao C , *et al.* Endoplasmic reticulum stress triggers an acute proteasome‐dependent degradation of ATF6. J Cell Biochem 2004; 92: 723–732. 15211570 10.1002/jcb.20118

[46] Vattem KM , Wek RC . Reinitiation involving upstream ORFs regulates ATF4 mRNA translation in mammalian cells. Proc Natl Acad Sci USA 2004; 101: 11269–11274. 15277680 10.1073/pnas.0400541101 PMC509193

[47] Wang Y , Shen J , Arenzana N , *et al.* Activation of ATF6 and an ATF6 DNA binding site by the endoplasmic reticulum stress response. J Biol Chem 2000; 275: 27013–27020. 10856300 10.1074/jbc.M003322200

[48] Lee K , Tirasophon W , Shen X , *et al.* IRE1‐mediated unconventional mRNA splicing and S2P‐mediated ATF6 cleavage merge to regulate XBP1 in signaling the unfolded protein response. Genes Dev 2002; 16: 452–466. 11850408 10.1101/gad.964702 PMC155339

[49] Thuerauf DJ , Marcinko M , Belmont PJ , *et al.* Effects of the isoform‐specific characteristics of ATF6α and ATF6β on endoplasmic reticulum stress response gene expression and cell viability. J Biol Chem 2007; 282: 22865–22878. 17522056 10.1074/jbc.M701213200

[50] Burton GJ , Yung HW , Cindrova‐Davies T , *et al.* Placental endoplasmic reticulum stress and oxidative stress in the pathophysiology of unexplained intrauterine growth restriction and early onset preeclampsia. Placenta 2009; 30(suppl A): S43–48. 19081132 10.1016/j.placenta.2008.11.003 PMC2684656

[51] Ohkuchi A , Hirashima C , Matsubara S , *et al.* Alterations in placental growth factor levels before and after the onset of preeclampsia are more pronounced in women with early onset severe preeclampsia. Hypertens Res 2007; 30: 151–159. 17460385 10.1291/hypres.30.151

[52] Romero R , Nien JK , Espinoza J , *et al.* A longitudinal study of angiogenic (placental growth factor) and anti‐angiogenic (soluble endoglin and soluble vascular endothelial growth factor receptor‐1) factors in normal pregnancy and patients destined to develop preeclampsia and deliver a small for gestational age neonate. J Matern Fetal Neonatal Med 2008; 21: 9–23. 18175241 10.1080/14767050701830480 PMC2587364

[53] Wikstrom AK , Larsson A , Eriksson UJ , *et al.* Placental growth factor and soluble FMS‐like tyrosine kinase‐1 in early‐onset and late‐onset preeclampsia. Obstet Gynecol 2007; 109: 1368–1374. 17540809 10.1097/01.AOG.0000264552.85436.a1

[54] Carmeliet P , Moons L , Luttun A , *et al.* Synergism between vascular endothelial growth factor and placental growth factor contributes to angiogenesis and plasma extravasation in pathological conditions. Nat Med 2001; 7: 575–583. 11329059 10.1038/87904

[55] Baker PN , Krasnow J , Roberts JM , *et al.* Elevated serum levels of vascular endothelial growth factor in patients with preeclampsia. Obstet Gynecol 1995; 86: 815–821. 7566855 10.1016/0029-7844(95)00259-T

[56] Drogat B , Auguste P , Nguyen DT , *et al.* IRE1 signaling is essential for ischemia‐induced vascular endothelial growth factor‐A expression and contributes to angiogenesis and tumor growth *in vivo* . Cancer Res 2007; 67: 6700–6707. 17638880 10.1158/0008-5472.CAN-06-3235

[57] Cindrova‐Davies T , Spasic‐Boskovic O , Jauniaux E , *et al.* Nuclear factor‐κB, p38, and stress‐activated protein kinase mitogen‐activated protein kinase signaling pathways regulate proinflammatory cytokines and apoptosis in human placental explants in response to oxidative stress: effects of antioxidant vitamins. Am J Pathol 2007; 170: 1511–1520. 17456758 10.2353/ajpath.2007.061035 PMC1854947

[58] Ichijo H. From receptors to stress‐activated MAP kinases. Oncogene 1999; 18: 6087–6093. 10557099 10.1038/sj.onc.1203129

[59] Urano F , Wang X , Bertolotti A , *et al.* Coupling of stress in the ER to activation of JNK protein kinases by transmembrane protein kinase IRE1. Science 2000; 287: 664–666. 10650002 10.1126/science.287.5453.664

[60] Yoshida H , Haze K , Yanagi H , *et al.* Identification of the *cis*‐acting endoplasmic reticulum stress response element responsible for transcriptional induction of mammalian glucose‐regulated proteins. Involvement of basic leucine zipper transcription factors. J Biol Chem 1998; 273: 33741–33749. 9837962 10.1074/jbc.273.50.33741

[61] Thuerauf DJ , Morrison LE , Hoover H , *et al.* Coordination of *ATF6*‐mediated transcription and ATF6 degradation by a domain that is shared with the viral transcription factor, VP16. J Biol Chem 2002; 277: 20734–20739. 11909875 10.1074/jbc.M201749200

[62] Di Iorio R , Marinoni E , Letizia C , *et al.* Adrenomedullin, a new vasoactive peptide, is increased in preeclampsia. Hypertension 1998; 32: 758–763. 9774376 10.1161/01.hyp.32.4.758

[63] Gratton RJ , Gluszynski M , Mazzuca DM , *et al.* Adrenomedullin messenger ribonucleic acid expression in the placentae of normal and preeclamptic pregnancies. J Clin Endocrinol Metab 2003; 88: 6048–6055. 14671210 10.1210/jc.2003-030323

[64] Hoffmann T , Penel C , Ronin C . Glycosylation of human prolactin regulates hormone bioactivity and metabolic clearance. J Endocrinol Invest 1993; 16: 807–816. 8144855 10.1007/BF03348932

[65] Yung HW , Hemberger M , Watson ED , *et al.* Endoplasmic reticulum stress disrupts placental morphogenesis: implications for human intrauterine growth restriction. J Pathol 2012; 228: 554–564. 22733590 10.1002/path.4068 PMC3532660

[66] Chung JY , Song Y , Wang Y , *et al.* Differential expression of vascular endothelial growth factor (VEGF), endocrine gland derived‐VEGF, and VEGF receptors in human placentas from normal and preeclamptic pregnancies. J Clin Endocrinol Metab 2004; 89: 2484–2490. 15126581 10.1210/jc.2003-031580 PMC3282114

[67] Groten T , Gebhard N , Kreienberg R , *et al.* Differential expression of VE‐cadherin and VEGFR2 in placental syncytiotrophoblast during preeclampsia – new perspectives to explain the pathophysiology. Placenta 2010; 31: 339–343. 20167365 10.1016/j.placenta.2010.01.014

[68] Kawakami T , Yoshimi M , Kadota Y , *et al.* Prolonged endoplasmic reticulum stress alters placental morphology and causes low birth weight. Toxicol Appl Pharmacol 2014; 275: 134–144. 24370435 10.1016/j.taap.2013.12.008

[69] Takahashi T , Shibuya M . The 230 kDa mature form of KDR/Flk‐1 (VEGF receptor‐2) activates the PLC‐γ pathway and partially induces mitotic signals in NIH3T3 fibroblasts. Oncogene 1997; 14: 2079–2089. 9160888 10.1038/sj.onc.1201047

