# Supplementary material for: Placental endoplasmic reticulum stress negatively regulates transcription of placental growth factor via ATF4 and ATF6β: implications for the pathophysiology of human pregnancy complications
Source: J Pathol. 2016 Jan 12;238(4):550–61. doi: 10.1002/path.4678 (PMC4784173; doi:10.1002/path.4678)
Supplement: Supplementary file 1 — Appendix S1. Supporting information [file PATH-238-550-s001.docx]

**Supporting information**

**Cell Culture**

Human choriocarcinoma JEG-3 cells were grown in RPMI 1640 medium (Invitrogen Ltd, Paisley, UK) supplemented with 10% heat-inactivated FBS (HI-FBS) (Invitrogen), penicillin (10,000U/ml), and streptomycin (10,000μg/ml) at 37°C in a 5% CO_2_ atmosphere. BeWo cells were cultured in DMEM/F-12 GlutaMAX TM (Invitrogen) with the same supplements and conditions.

**Western blot analysis**

Western blots were performed on both cell lysates and culture media to quantify relative levels and phosphorylation of specific proteins. The procedures were described in detail in a previous study [37].

Conditioned media were collected and centrifuged at 4000 g for 30 min at 4°C to remove cell debris before concentrating using Vivaspin20-10K (Sartorius Stedim Biotech, UK) for analysis of released PlGF. Concentrated samples were stored at −80°C until further analysis. Equal volumes of conditioned media were used from each sample.

***In Vitro* H/R Experiments**

For the hypoxia-reoxygenation (H/R) challenge, BeWo and JEG-3 cells were cultured in normal growth medium with 10% FBS in an hypoxic incubator (Ex Vivo; BioSpherix, Lacona, NY, USA). Cells were under a 6 h repetitive cycle between 1% and 10% O_2_ for 24 h. Control cells were incubated at either 20% or 10% O_2_ under standard culture conditions.

**RNA isolation, reverse transcription polymerase chain reaction (RT-PCR) and Quantitative real-time RT-PCR analysis**

Total RNA was isolated by RNeasy Mini Kit (QIAGEN) according to the manufacturer’s instructions. The RNA concentration was determined using the NanoDrop 1000 system (Thermo Scientific). Total RNA (1 μg) was used for reverse transcription in 20 μl reaction volumes using SuperScript II RTase (Invitrogen, UK) according to manufacturer’s protocol. For quantitative RT-PCR (qPCR), SYBR Green JumpStart qPCR MasterMix (Sigma, UK) was mixed with the first strand cDNA template and appropriate primer pairs using Applied Biosystems 7500 Fast Real-Time PCR System (Life Technologies, UK) according to the manufacturer's protocols. The thermo-cycling parameters for PCR amplification were: 5 min at 95°C followed by 40 cycles of 15 s at 95°C and 1 min at 60°C. The specificity of the amplification was determined by melting curve analysis (55°C-95°C, held and read every 10 s at each 0.5°C increment). This showed a sharp and single amplified product for each reaction. Delta Ct values for target genes were calculated from the mean Ct values of two housekeeping genes Glyceraldehyde 3-phosphate dehydrogenase (GAPDH) and Tata box binding protein (TBP). RNA concentration was calculated by the ΔΔCt method of relative quantification. Each cDNA sample was analyzed in triplicate.

| **Gene** |  | **Primer Sequence, 5’-3’** | **Amplicon Size** |
| --- | --- | --- | --- |
| *PlGF* | Sense | TGATCTCCCCTCACACTTTGC | 62 bp |
|  | Antisense | CACCTTGGCCGGAAAGAA |  |
| *ATF4* | Sense | GACGGAGCGCTTTCCTCTT | 69 bp |
|  | Antisense | TCCACAAAATGGACGCTCAC |  |
| *ATF6α* | Sense | CTCCGAGATCAGCAGAGGAA | 81bp |
|  | Antisense | AATGACTCAGGGATGGTGCT |  |
| *ATF6β* | Sense | GAGGTGCTCCATGTGAAGACA | 85bp |
|  | Antisense | GGACGGTTTCAAATGAGGATGTT |  |
| *XBP1s* | Sense | CCCTCCAGAACATCTCCCCAT | 101bp |
|  | Antisense | ACATGACTGGGTCCAAGTTGT |  |
| *GAPDH* | Sense | CGCTCTCTGCTCCTCCTGTT | 81bp |
|  | Antisense | CCATGGTGTCTGAGCGATGT |  |
| *TBP* | Sense | GGGTTTTCCAGCTAAGTTCTTG | 137bp |
|  | Antisense | CTGTAGATTAAACCAGGAAATAAC |  |

**siRNA knockdown experiment**

BeWo cells (~40% confluent) were transfected with non-targeting control small interfering RNAs (control siRNA) (5 nM) and target gene siRNA 5 nM using the Lipofectamine RNAiMAX (Life Technologies) transfection reagent, according to the manufacturer’s instructions. After 48 h of transfection, the medium containing siRNA and transfection reagents was removed and cells were treated in the presence or absence of thapsigargin (100nM) in serum-free medium for 24 h. Efficiency of knockdown of target transcripts was evaluated using quantitative RT-PCR analysis and presented in Supplementary Figure 2. siRNAs for ATF4 (SASI_Hs02_00332313, NM_001675) and XBP-1s (EHU069131) were purchased from Sigma. siRNA for ATF6α (M-009917-01-0010) and ATF6β (M-00805-00-0005) were siGenome smartPool from Dharmacon.

**Immunohistochemistry**

Paraformaldehyde-fixed tissues embedded in paraffin wax were sectioned at 7µm. After dewaxing and blocking of endogenous peroxidases by incubation with 3 % H_2_O_2_ for 30 min, antigen retrieval was performed. For ATF4, ATF6α and ATF6β this involved Tris-EDTA (TE) buffer (10 mM Tris (pH 8), 1 mM EDTA, 0.05% Tween) in a pressure cooker for 2 min, whereas for PlGF proteinase K (20μg/ml) was used in TE buffer, pH8 at room temperature, for 30 min. The sections were incubated with non-immune serum for 30 min, followed by incubation with primary antibodies, PlGF (1/50; Santa Cruz Biotechnology, sc-1880), phospho-IRE1α (1/100; Abcam, ab48187), ATF4 (1/250; Abcam, ab31390), ATF6α (1/600; Abcam, ab37149), ATF6β (1/100; Novus Biologicals, NBP1-91687) overnight at 4°C. After washing, the slides were incubated with biotinylated anti-mouse or anti-rabbit secondary antibodies (Vector Laboratories Ltd). Binding was detected using Vectastain Elite ABC kits (Vector Laboratories, Peterborough, UK) and SigmaFast DAB (Sigma, Poole, UK), according to the manufacturers’ instructions. Sections were lightly counterstained with haematoxylin. Negative controls were performed by omitting primary antibodies.
