# Supplementary figures and images for: Placental endoplasmic reticulum stress negatively regulates transcription of placental growth factor via ATF4 and ATF6β: implications for the pathophysiology of human pregnancy complications
Source: J Pathol. 2016 Jan 12;238(4):550–61. doi: 10.1002/path.4678 (PMC4784173; doi:10.1002/path.4678)

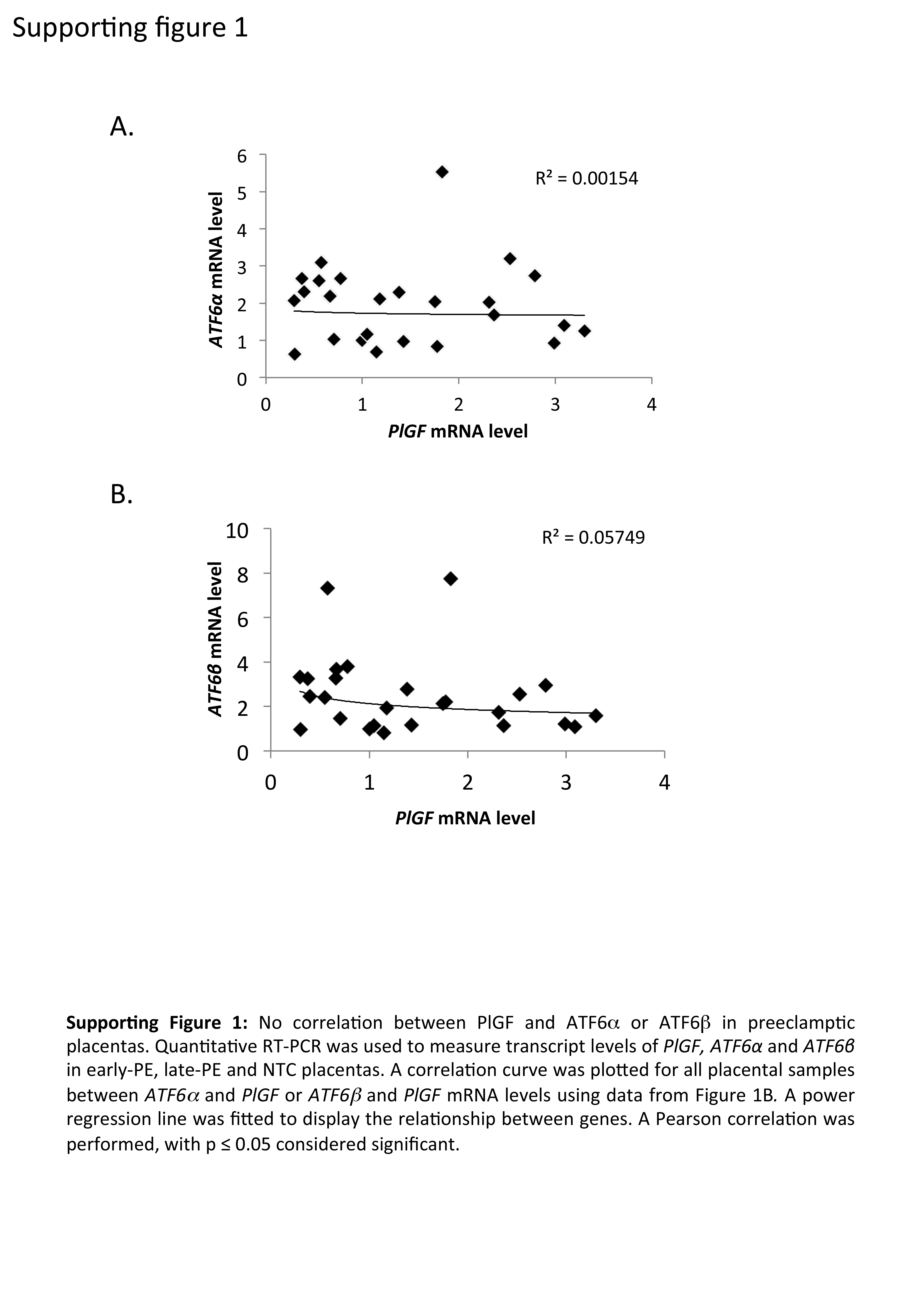

Supplement: Supplementary file 2 — Figure S1 No correlation was found between PlGF and ATF6α or ATF6β in pre‐eclamptic placentae. qPCR was used to measure transcript levels of PlGF, ATF6α and ATF6β in early‐PE, late‐PE and NTC placentae; a correlation curve was plotted for all placental samples between ATF6α and PlGF or ATF6β and PlGF mRNA levels, using data from Figure 1B. A power regression line was fitted to display the relationship between genes. Pearson correlation was performed, with p ≤ 0.05 considered significant [file PATH-238-550-s002.tif]

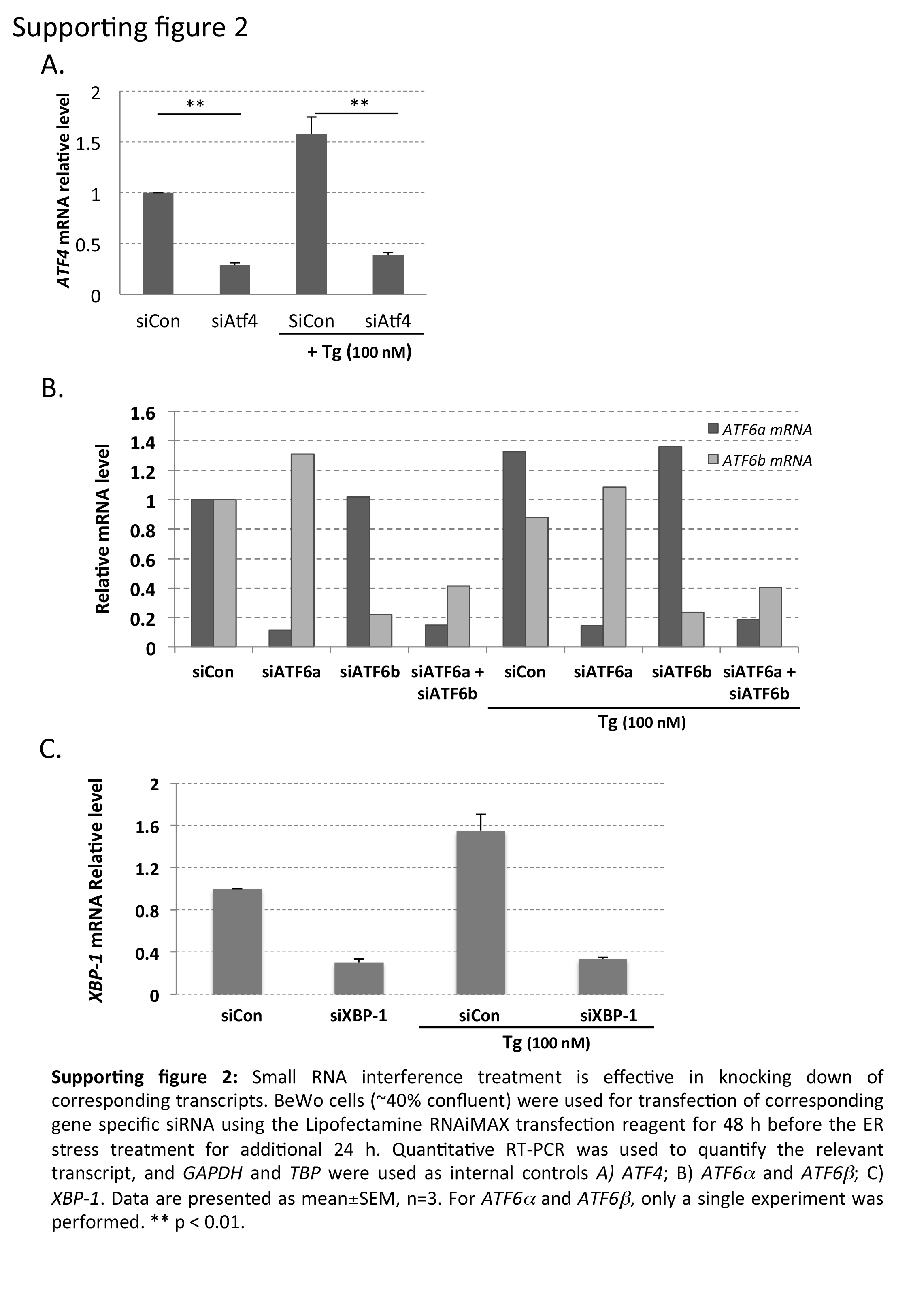

Supplement: Supplementary file 3 — Figure S2 Small RNA interference treatment is effective in knocking down corresponding transcripts. BeWo cells (∼40% confluent) were used for transfection of corresponding gene‐specific siRNA, using the Lipofectamine RNAiMAX transfection reagent for 48 h before the ER stress treatment for an additional 24 h. (A, C, D) qPCR was used to quantify the relevant transcript and GAPDH and TBP were used as internal controls: (A) ATF4; (B) ATF6α and ATF6β; (C) XBP‐1; data are presented as mean ± SEM, n = 3; for ATF6α and ATF6β, only a single experiment was performed; **p < 0.01 [file PATH-238-550-s003.tif]
