# Supplementary material for: Placental endoplasmic reticulum stress negatively regulates transcription of placental growth factor via ATF4 and ATF6β: implications for the pathophysiology of human pregnancy complications
Source: J Pathol. 2016 Jan 12;238(4):550–61. doi: 10.1002/path.4678 (PMC4784173; doi:10.1002/path.4678)
Supplement: Supplementary file 4 — Table S1 The primer sequences used for quantitative real‐time RT‐PCR and their corresponding amplicon sizes. [file PATH-238-550-s004.doc]

**Table S1.** The primer sequences used for quantitative real-time RT-PCR and their corresponding amplicon sizes.

| **Gene** |  | **Primer sequence, 5**′–**3**′ | **Amplicon size (bp)** |
| --- | --- | --- | --- |
| *PlGF* | Sense | TGATCTCCCCTCACACTTTGC | 62 |
|  | Antisense | CACCTTGGCCGGAAAGAA |  |
| *ATF4* | Sense | GACGGAGCGCTTTCCTCTT | 69 |
|  | Antisense | TCCACAAAATGGACGCTCAC |  |
| *ATF6α* | Sense | CTCCGAGATCAGCAGAGGAA | 81 |
|  | Antisense | AATGACTCAGGGATGGTGCT |  |
| *ATF6β* | Sense | GAGGTGCTCCATGTGAAGACA | 85 |
|  | Antisense | GGACGGTTTCAAATGAGGATGTT |  |
| *XBP1s* | Sense | CCCTCCAGAACATCTCCCCAT | 101 |
|  | Antisense | ACATGACTGGGTCCAAGTTGT |  |
| *GAPDH* | Sense | CGCTCTCTGCTCCTCCTGTT | 81 |
|  | Antisense | CCATGGTGTCTGAGCGATGT |  |
| *TBP* | Sense | GGGTTTTCCAGCTAAGTTCTTG | 137 |
|  | Antisense | CTGTAGATTAAACCAGGAAATAAC |  |
